# Using Digital Phenotyping to Discriminate Unipolar Depression and Bipolar Disorder: Systematic Review

**DOI:** 10.2196/72229

**Published:** 2025-05-23

**Authors:** Rongrong Zhong, XiaoHui Wu, Jun Chen, Yiru Fang

**Affiliations:** 1 Clinical Research Center & Division of Mood Disorders Shanghai Mental Health Center Shanghai Jiao Tong University School of Medicine Shanghai China; 2 Department of Psychiatry & Affective Disorders Center Ruijin Hospital Shanghai Jiao Tong University School of Medicine Shanghai China; 3 College of Psychiatry and Clinical Psychology, Shanghai Jiao Tong University School of Medicine Shanghai China; 4 Shanghai Key Laboratory of Psychotic disorders Shanghai China

**Keywords:** digital phenotyping, depression, bipolar disorder, smartphone, wearable, audiovisual, multimodal, artificial intelligence, AI

## Abstract

**Background:**

Differentiating bipolar disorder (BD) from unipolar depression (UD) is essential, as these conditions differ greatly in their progression and treatment approaches. Digital phenotyping, which involves using data from smartphones or other digital devices to assess mental health, has emerged as a promising tool for distinguishing between these two disorders.

**Objective:**

This systematic review aimed to achieve two goals: (1) to summarize the existing literature on the use of digital phenotyping to directly distinguish between UD and BD and (2) to review studies that use digital phenotyping to classify UD, BD, and healthy control (HC) individuals. Furthermore, the review sought to identify gaps in the current research and propose directions for future studies.

**Methods:**

We systematically searched the Scopus, IEEE Xplore, PubMed, Embase, Web of Science, and PsycINFO databases up to March 20, 2025. Studies were included if they used portable or wearable digital tools to directly distinguish between UD and BD, or to classify UD, BD, and HC. Original studies published in English, including both journal and conference papers, were included, while reviews, narrative reviews, systematic reviews, and meta-analyses were excluded. Articles were excluded if the diagnosis was not made through a professional medical evaluation or if they relied on electronic health records or clinical data. For each included study, the following information was extracted: demographic characteristics, diagnostic criteria or psychiatric assessments, details of the technological tools and data types, duration of data collection, data preprocessing methods, selected variables or features, machine learning algorithms or statistical tests, validation, and main findings.

**Results:**

We included 21 studies, of which 11 (52%) focused on directly distinguishing between UD and BD, while 10 (48%) classified UD, BD, and HC. The studies were categorized into 4 groups based on the type of digital tool used: 6 (29%) used smartphone apps, 3 (14%) used wearable devices, 11 (52%) analyzed audiovisual recordings, and 1 (5%) used multimodal technologies. Features such as activity levels from smartphone apps or wearable devices emerged as potential markers for directly distinguishing UD and BD. Patients with BD generally exhibited lower activity levels than those with UD. They also tended to show higher activity in the morning and lower in the evening, while patients with UD showed the opposite pattern. Moreover, speech modalities or the integration of multiple modalities achieved better classification performance across UD, BD, and HC groups, although the specific contributing features remained unclear.

**Conclusions:**

Digital phenotyping shows potential in distinguishing BD from UD, but challenges like data privacy, security concerns, and equitable access must be addressed. Further research should focus on overcoming these challenges and refining digital phenotyping methodologies to ensure broader applicability in clinical settings.

**Trial Registration:**

PROSPERO CRD42024624202; https://www.crd.york.ac.uk/PROSPERO/view/CRD42024624202

## Introduction

### Background

Mood disorders are a highly prevalent and recurrent group of mental disorders associated with a substantial risk of suicide [1], primarily encompassing unipolar depression (UD) and bipolar disorder (BD) [2]. Approximately 1 in 4 individuals is estimated to experience an affective disorder at least once in their lifetime, often leading to substantial and lasting disability for those affected [3]. UD is primarily characterized by substantial and persistent low mood. In contrast, BD involves manic or hypomanic episodes (elevated mood, racing thoughts, and increased activity) and depressive episodes (low mood, slowed thinking, and reduced activity). Both disorders are marked by enduring mood changes impacting emotional, cognitive, and behavioral domains [4]. However, the course of BD often begins with depressive episodes, leading to a substantial risk of misdiagnosis, as approximately 40% to 69% of patients with BD are initially diagnosed with UD [5,6]. This misdiagnosis can have serious consequences, including inappropriate medication prescriptions, triggering manic episodes, prolonged illness duration, increased risk of recurrence, heightened suicide risk, and an overall poorer response to treatment [5,7-10].

Currently, psychiatrists typically diagnose based on established criteria (such as the *Diagnostic and Statistical Manual of Mental Disorders, Fifth Edition* and the *International Classification of Diseases, Eleventh Revision*), relying on one-time self-reports from patients and their families. This approach heavily depends on the clinician’s experience and often lacks continuity and objectivity. In contrast, digital phenotyping offers a promising solution, with this concept being a groundbreaking advancement in the field, first introduced in 2015 [11]. Digital phenotyping includes long-term active data (eg, participants completing daily self-assessment questionnaires via smartphone apps) [12], providing clinicians with a more comprehensive and continuous flow of information. It also offers objective data based on digital devices, such as physiological measurements (eg, skin temperature, heart rate, blood volume pulse, and electrodermal activity) and behavioral indicators (eg, acoustic features, gestures, and facial expressions) [13].

Compared to traditional diagnostic methods, digital phenotyping has great potential to improve diagnostic accuracy and timeliness. However, it generates vast amounts of data that require more robust processing and analysis. To address this, leveraging artificial intelligence, such as machine learning, in mental health is essential. Currently, machine learning has gradually emerged as a powerful tool for exploring high-dimensional and real-time data associated with digital phenotyping. It provides an opportunity to “make sense” of these digital phenotypes and the realities they attempt to represent in the context of mental health [14-16]. Some believe this technology has the potential to offer deeper insights into the neurobiological mechanisms underlying psychiatric disorders [17]. It may also facilitate the development of new transdiagnostic models for understanding symptoms [12]. This aligns with the “research domain criteria” perspective proposed in recent years [18]. In addition to classification tasks, machine learning algorithms may also have the capability to predict episodes or even suicidal risk, enabling clinicians to make more accurate and timely clinical decisions. Consequently, digital phenotyping, supported by machine learning, has carved out an important role in psychiatry [19], helping clinicians access individualized behavioral, emotional, and other data from patients with mental disorders. This approach not only enhances psychiatrists’ understanding of symptoms and the disorders themselves [20,21], but may also compensate for the current lack of reliable biomarkers.

### Objectives

In this study, we conducted a systematic review of original articles from both journals and conference proceedings, exploring the use of portable or wearable digital tools for distinguishing UD and BD, as well as classifying UD, BD, and healthy control (HC) individuals. We examined the studies by considering factors such as demographic characteristics and the diagnostic criteria or psychiatric assessments used. In addition, we analyzed the technological tools and data types used, the duration of data collection, data preprocessing methods, selected variables or features, computational techniques applied, validation approaches, and the results achieved.

This review is essential because many previous studies in this field have either focused on specific tools or were limited by small sample sizes, which restricts their applicability to broader populations. Furthermore, the lack of consistency in methodologies and the absence of comprehensive validation across different groups have hindered the generalizability of the findings. This review aimed to address these limitations and provide valuable insights into the potential of digital phenotyping for more accurate and reliable differentiation between these groups.

## Methods

### Information Sources and Search Strategy

This systematic review was conducted in agreement with the PRISMA (Preferred Reporting Items for Systematic reviews and Meta-Analyses) guidelines (Multimedia Appendix 1) [22] and is listed in the PROSPERO register (CRD42024624202). While the review was generally conducted according to the registered protocol, some minor deviations occurred during the study. These adjustments primarily involved refining and clarifying the study objectives and title to better reflect the focus of the review. In addition, we expanded the scope to include studies that classified UD, BD, and HC, which was not initially specified in the protocol. Finally, we excluded studies based on nonclinically diagnosed data, such as those relying on social media samples, to ensure that only studies adhering to professional diagnostic standards were included.

We conducted a comprehensive search across 6 major databases, including Scopus, IEEE Xplore, PubMed, Embase, Web of Science, and PsycINFO, for articles published up to March 20, 2025. The search terms used in each database are provided in Multimedia Appendix 2. We included original studies, both journal articles and conference papers, published in English, with no restrictions on publication date.

### Eligibility Criteria

Eligible studies involved participants diagnosed with UD, BD, or HC and used portable or wearable digital devices such as smartphone apps, wearable sensors, or audio or visual recordings. The studies were required to either compare digital phenotyping results with diagnostic outcomes from professional medical evaluations, compare UD with BD, or perform a classification task involving UD, BD, and HC. The primary outcome of interest was classification performance metrics such as sensitivity, specificity, area under the curve (AUC), accuracy, recall, and precision, but studies reporting *t* tests, ANOVA, nonparametric tests, or correlation analyses were also considered.

Following the PICOS (population, intervention, comparison, outcome, and study design) criteria, we excluded studies that were reviews, meta-analyses, or written in languages other than English. In addition, studies were excluded if they used technologies unsuitable for daily monitoring, were based on electronic health records or clinical data, or had diagnoses not made through professional medical evaluations (eg, studies based on social media data).

### Selection Process

#### Title and Abstract Selection

The titles and abstracts of all articles that matched the search criteria were double-screened. After reviewing them, we excluded studies that did not address the research question.

#### Full-Text Selection

We included in the systematic review only papers that aimed to differentiate between UD and BD, either directly or by classifying UD, BD, and HC, using digital phenotyping, according to the previously defined PICOS criteria. The included papers were read thoroughly to extract the relevant data.

All articles that met the search criteria were independently screened by 2 reviewers (RZ and XW) during both the title and abstract selection and the full-text selection stages. In cases of disagreement, a third reviewer (YF) was consulted to achieve consensus.

#### Data Extraction

For each study, the following information was extracted: geographic region; population; epidemiological data of the sample (number and percentage of females and mean age); diagnostic criteria or psychiatric assessments; type of technology and data collected; duration of data collection; data preprocessing methods; specific variables or features selected; machine learning algorithms or statistical tests used; validation; and main findings. Data extraction was independently conducted by 2 authors (RZ and XW). Discrepancies were resolved through discussion or with the involvement of a third author (YF).

### Synthesis Method

The included papers were grouped based on the type of digital tool used (smartphone apps, wearable devices, audiovisual recordings, or multimodal technologies) and the comparison type (UD vs BD or UD vs BD vs HC). A narrative synthesis approach was used to summarize and compare study characteristics, data modalities, analytical methods, and classification performance. Quantitative pooling was not performed due to the heterogeneity in study designs and feature selection.

### Study Risk of Bias Assessment

The quality of the selected studies was independently evaluated by 2 reviewers (RZ and XW) using the Quality Assessment of Diagnostic Accuracy Studies-2 (QUADAS-2) [23] tool. Discrepancies were resolved through discussion or with the involvement of a third author (YF). QUADAS-2 comprises 4 key domains: patient selection, index test, reference standard, and flow and timing. For each domain, the risk of bias and concerns regarding applicability were assessed and categorized as low, high, or unclear risk.

## Results

### Overview

**Figure 1:**
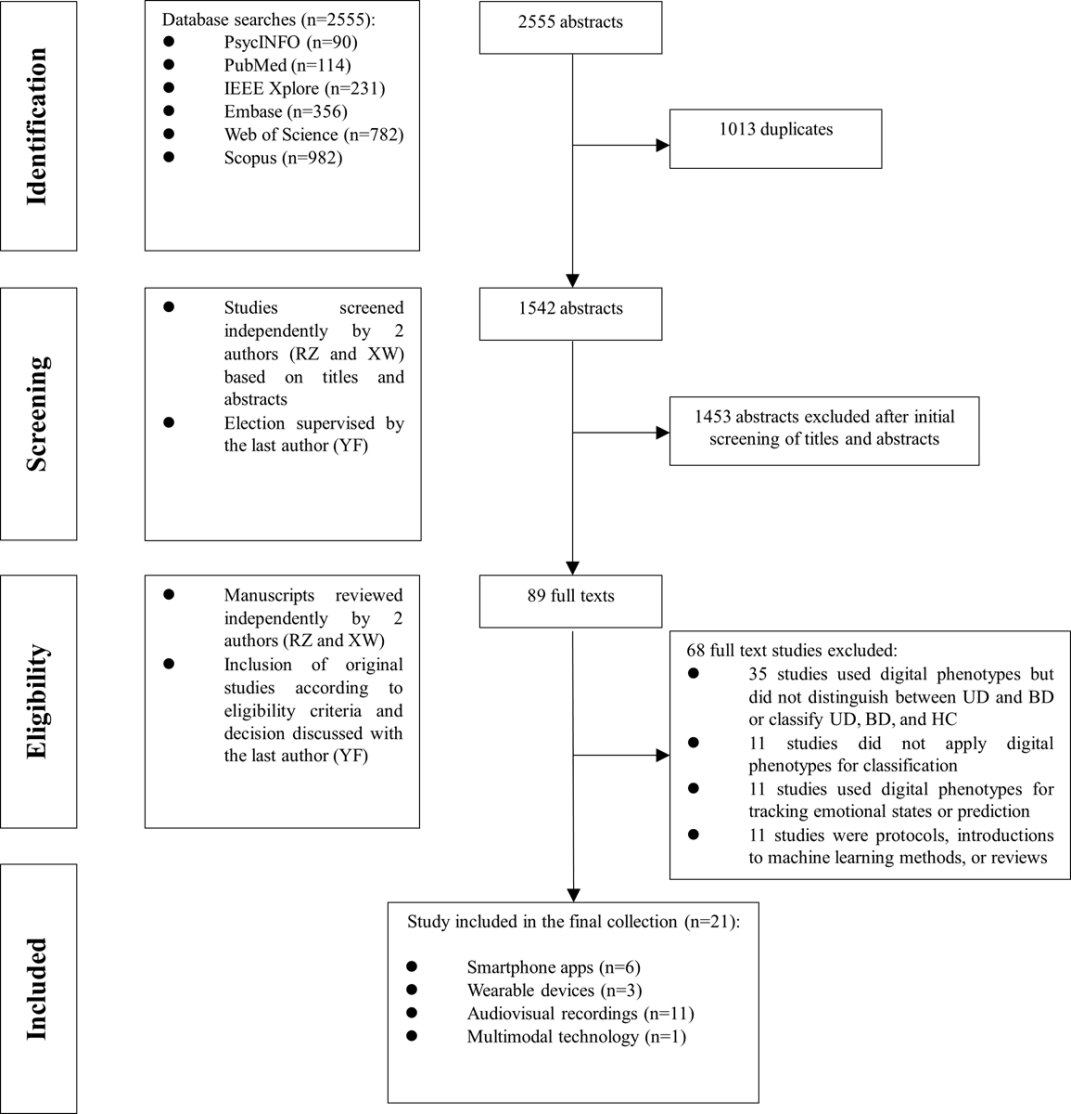
Flowchart of review process and study selection. BD: bipolar disorder; HC: healthy control; UD: unipolar depression.

As shown in Figure 1, a thorough search was conducted across 6 major databases, yielding a total of 2555 articles. Specifically, PsycINFO identified 90 articles, PubMed provided 114 articles, IEEE Xplore returned 231 articles, Embase found 356 articles, Web of Science collected 782 articles, and Scopus gathered 982 articles. After removing 1013 duplicate records, the titles and abstracts of the remaining 1542 articles were screened for relevance to the topic, narrowing the selection to 89 articles for full-text eligibility assessment. During the eligibility screening, 68 full-text studies were excluded after a detailed evaluation. Among these, 35 studies applied digital phenotyping but did not distinguish between UD and BD or classify UD, BD, and HC. In total, 11 studies did not use digital phenotyping at all. Another 11 studies used digital phenotyping to track emotional states or make predictions. The remaining 11 studies were either protocols, introductions to machine learning methods, or reviews. These exclusions ensured that the final selection aligned precisely with the study’s specific objectives. The final sample comprised 21 articles, with the earliest study dating back to 2016, emphasizing the novelty of this research topic. Of the 21 studies, 11 (52%) studies directly distinguished between UD and BD (Table 1 and Multimedia Appendix 3 [24-44]), while 10 (48%) studies classified UD, BD, and HC (Table 2 and Multimedia Appendix 3). It is important to note that studies based on the RADMIS trials or those using the CHI-MEI mood disorder database may have partly overlapping populations. However, they were not excluded due to their very different methods and results. In addition, although studies using the CHI-MEI database did not explicitly report the diagnostic criteria or psychiatric assessments, data collection was carried out in collaboration with clinicians at Chi Mei Medical Centre. Consequently, these studies were not excluded.

**Table 1:** Methodological details of included studies that directly distinguish between UD^a^ and BD^b^.

Study		Data recording	Data used	Data preprocessing	Specific variable or feature selection	Machine learning models or statistic test	Validation	Findings
**Smartphone apps**								
	Faurholt-Jepsen et al [24], 2022	6 mo	Active data (self-assessments) and passive data (location information) with a smartphone app (Monsenso system)	Minimum of 50 location samples per day; exclusion of points with unrealistic acceleration	Stops, moves, places, routine index, radius of gyration, and location entropy	BBC^c^	10-fold stratified cross-validation	Discrimination between patients with UD and patients with BD (based on passive data): UD vs BD, overall: AUC^d^=0.75; UD vs BD, euthymic state: AUC=0.79; UD vs BD, depressive state: AUC=0.79
	Faurholt‐Jepsen et al [25], 2022	1-972 d	Naturalistic phone calls, voice collected from a smartphone app (Monsenso system)	Removal of voice data without a corresponding patient-reported smartphone-based data entry of either mood, activity, or sleep	Acoustic features such as pitch, loudness, and energy	RF^e^	5-fold cross-validation	Discrimination between patients with UD and patients with BD: UD vs BD: ACC^f^=0.73; AUC=0.58; sensitivity=0.27; specificity=0.84; UD vs BD, euthymia: ACC=0.76; AUC=0.43; sensitivity=0.18; specificity=0.79; UD vs BD, depression: ACC=0.66; AUC=0.48; sensitivity=0.16; specificity=0.81
	Langholm et al [26], 2023	12 wk	Active data (self-reported survey) and passive data (user activity, geolocation, motion, exercise, and device rotation) with a smartphone app (mindLAMP)	Exclusion of bins with data quality <0.8 and imputation of missing values using mean feature values	Home time, entropy, sleep duration, screen duration, and survey scores	SVM^g^, LR^h^, and DT^i^	Repeated (n=3); stratified K-fold (*k*=5) cross-validation	Binary classification results of UD vs BD: all features: ACC=57.1%; AUC=0.61; active features: ACC=64.3%; AUC=0.62; passive features: ACC=50%; AUC=0.52
	Faurholt-Jepsen et al [27], 2023	6 mo	Active data (self-assessments; Monsenso system)	Missing at random for missing data	Patient-reported daily evaluations about irritability, mood, activity, sleep, stress, and anxiety	Mixed-effects regression models	N/A^j^	Patients with UD spent a higher proportion of time with the presence of irritability compared with patients with BD (depressive state)
	Faurholt-Jepsen et al [28], 2024	6 mo	Active data (self-assessments) and passive data (phone calls, text messages, and screen) with a smartphone app (Monsenso system)	Removal of entire day’s data without a single reading for a day and imputation techniques for missing data	Number or duration of phone calls, text messages, and screen use	RF	Leave-one-patient-out cross-validation	Discrimination between patients with UD and patients with BD (based on passive data): UD vs BD, overall: AUC=0.48; sensitivity=0.54; specificity=0.44; UD vs BD, euthymia: AUC=0.46; sensitivity=0.69; specificity=0.21; UD vs BD, depressive state: AUC=0.42; sensitivity=0.30; specificity=0.60
	Faurholt-Jepsen et al [29], 2025	6 mo	Active data (self-assessments; Monsenso system)	Missing at random for missing data	Patient-reported daily mood and activity scales	Linear mixed-effects regression models	N/A	Patients with BD presented with a lower level of activity as compared with patients with UD (overall, euthymic state and depressive state); there were no differences in mood and activity instability between the 2 groups
**Wearable devices**								
	Tanaka et al [30], 2018	3 wk	Daytime activity data with wearable activity trackers (Actiwatch)	Exclusion of participants with missing values (days with zero activity across all epochs)	5 principal components such as the total amount of activity and the activity ratio	PCA^k^	N/A	Several temporal patterns of intraday activities were associated with the differences between UD and BD: BD showed a high activity pattern in the morning and a low activity pattern in the evening; UD showed a low activity pattern in the morning and a high activity pattern in the evening
**Audiovisual recordings**								
	Yang et al [31], 2016	1 d	Facial expressions and speech responses from interviews with a clinician after participants watched 6 videos	Application of a domain adaptation method called hierarchical spectral clustering–based denoising autoencoder	Emotion profiles, action units, 384-dimensional acoustic features and 98-dimensional facial feature vector	HMM^l^ and CHMM^m^	13-fold cross-validation	Classification of patients with UD vs patients with BD: optimal ACC=65.38%
	Horigome et al [32], 2020	—^n^	Body motion with a red-green-blue-depth sensor	Nonlinear fitting with smoothing spline; splitting data into smaller segments for missing data >5 seconds	7 types of features, such as position, speed, acceleration, and jerk, at 4 body joints	*χ*^2^ test; 1-way ANOVA; and Tukey honestly significant difference test	N/A	No significant difference in any head motion features between the UD and BD groups
	Yamamoto et al [33], 2020	10 min	Nonstructured interviews (nonspecific topics) with a research psychiatrist or psychologist	Exclusion of overlapping voice frames and outlier data using IQR	Speech rate, pause time, and response time	ANCOVA^o^	N/A	Patients with UD showed longer response time than patients with BD, but there were no significant differences in speech rate and pause time
	Pan et al [34], 2023	—	Vocal features collected from 4 vocal tasks: video watching, text reading, question answering, and picture description	Exact gender matching using random sampling; case-control matching within classification tasks	Mel-frequency cepstral coefficients	LR	5-fold cross-validation	Classification of patients with UD vs patients with BD: ACC=0.50; AUC=0.50

^a^UD: unipolar depression.

^b^BD: bipolar disorder.

^c^BBC: balanced bagging classifier.

^d^AUC: area under the curve.

^e^RF: random forest.

^f^ACC: accuracy.

^g^SVM: support vector machine.

^h^LR: logistic regression.

^i^DT: decision tree.

^j^N/A: not applicable.

^k^PCA: principal component analysis.

^l^HMM: hidden Markov model.

^m^CHMM: coupled hidden Markov model.

^n^Not available.

^o^ANCOVA: analyses of covariance.

**Table 2:** Methodological details of included studies that classify UD^a^, BD^b^, and HC^c^.

Study			Data recording		Data used		Data preprocessing		Specific variable or feature selection		Machine learning models or statistic test		Validation		Findings
**Wearable devices**															
	Anmella et al [35], 2023	2 d		Physiological data with wearable devices (Empatica E4)		Rules-based filter for invalid physiological data and time unit set to 1 second		X, Y, or Z-axis acceleration, blood volume pulse, electrodermal activity, heart rate, and skin temperature		BiLSTM^d^		—^e^		7-class classification task: ACC^f^=0.7; AUROC^g^=0.69; *F*_1_-score=0.6927	
	Zakariah and Alotaibi [36], 2023	5-20 d		General levels of activity with a wearable Actiwatch		Imputation techniques (mean imputation, median imputation, or regression-based imputation) for missing values; transformed categorical variables into numerical representations		Motor activity measurement from the Actiwatch		UMAP^h^ and NN^i^		Leave-one-out validation		4-class classification task: ACC=0.991; *F*_1_-score=0.9887	
**Audiovisual recordings**															
	Yang et al [37], 2016	1 d		Speech responses from 5 questions after participants watched 6 videos		Silence removal and speech segmentation based on energy and spectral centroid as features for threshold definition		Emotion profiles, 39 dimensions of Mel-frequency cepstral coefficients and acoustic features of 384 dimensions		SVM^j^, MLP^k^, LSTM^l^, and BiLSTM		13-fold cross-validation		3-class classification task: optimal ACC=76.92%	
	Su et al [38], 2017	1 d		Facial expressions elicited by 6 emotional video clips		Select a time interval and segment each facial image into 12 mutually independent facial regions		8 basic orientations of motion vector in microscopic facial expression		HMM^m^ and LSTM		12-fold cross-validation		3-class classification task: optimal ACC=67.7%	
	Hong et al [39], 2018	1 d		Facial expressions elicited by 6 emotional video clips		Select time interval and facial points were aligned to a new coordinate		12 action units		MLP, SVM, GMM^n^, and LSTM		12-fold cross-validation		3-class classification task: optimal ACC=61.1%	
	Huang et al [40], 2019	1 d		Speech responses from interviews with a clinician after participants watched 6 videos		Use of hierarchical spectral clustering algorithm for database adaptation		Emotion profiles and 32-dimensional acoustic features		SVM, CNN^o^, and LSTM		Leave-one cross-validation		3-class classification task: optimal ACC=75.56%	
	Su et al [41], 2020	1 d		Facial expressions and speech responses from interviews with a clinician after participants watched 6 videos		Hierarchical spectral clustering and denoising autoencoder method for database adaptation		Emotion profiles, action units, 384 acoustic features and 49 facial expression feature points		SVM, HMM, MLP, GRU^p^, CNN, RNN^q^, and LSTM		13-fold cross-validation		3-class classification task: optimal ACC=76.9%	
	Hong et al [42], 2021	1 d		Facial expressions elicited by 6 emotional video clips		Selection of four 4-second intervals per elicitation video based on the facial expression intensity of all participants		Action units for macroscopic facial expressions and motion vectors for microscopic facial expressions		MLP, NN, and LSTM		12-fold cross-validation		3-class classification task: optimal ACC=72.2%	
	Luo et al [43], 2024	1 d		Voice signals collected from 7 pieces of reading material		Power normalization and speech segmentation into 7 parts corresponding to the 7 reading materials		120 vocal features for classification, such as the mean value of root-mean-square energy		DT^r^, NB^s^, SVM, KNN^t^, EL^u^, and CNN		—		3-class classification task: optimal ACC=95.6%	
**Multimodal technology**															
	Wu et al [44], 2024	1 d		Text, audio, facial attributes, heart rate, and eye movement with mobile devices while participants have a conversation with a virtual assistant		—		Word embedding; 5 spectral features, facial attribute embedding, 23 heart rate variability indices, and 7 eye movement features (fixation and saccade)		RF^v^, LSTM, and DT		5-fold cross-validation		5-class classification task: optimal ACC=90.26%	

^a^UD: unipolar depression.

^b^BD: bipolar disorder.

^c^HC: healthy control.

^d^BiLSTM: bidirectional long short-term memory.

^e^Not available.

^f^ACC: accuracy.

^g^AUROC: area under the receiver operating characteristic.

^h^UMAP: uniform manifold approximation and projection.

^i^NN: neural network.

^j^SVM: support vector machine.

^k^MLP: multilayer perceptron.

^l^LSTM: long short-term memory.

^m^HMM: hidden Markov model.

^n^GMM: Gaussian mixture model.

^o^CNN: convolutional neural network.

^p^GRU: gated recurrent unit.

^q^RNN: recurrent neural network.

^r^DT: decision tree.

^s^NB: naive Bayes.

^t^KNN: k-nearest neighbor.

^u^EL: ensemble learning.

^v^RF: random forest.

### Synthesized Findings

#### Overview

In this subsection, we present the key findings relevant to this systematic review for each study. The studies are categorized according to the type of digital devices used, with some also detailing the clinical staging of UD or BD. Audio and visual recordings were the most commonly used tools in this area, used in 11 (52%) out of 21 studies. In addition, 3 (14%) out of 21 studies used wearable devices, while 6 (29%) used smartphone apps to collect digital phenotyping data for distinguishing between UD and BD. Only 1 (5%) out of 21 studies used a multimodal approach, collecting diverse information including text, audio, facial expressions, heart rate, and eye movement during participant interactions with a virtual assistant. This study was classified as “multimodal technology.” Among the 11 (52%) out of 21 studies that directly distinguished between UD and BD, 8 (73%) described the mood states of patients (depressive, manic, mixed, or euthymic state). Of these 8 studies, 5 (63%) studies classified depressive and euthymic states based on participants’ self-reported scores [24,25,27-29], while the other 3 (38%) studies assessed mood states through general clinical interviews [30], Hamilton Depression Scale and Young Mania Rating Scale [32], or confirmation of clinical staging by clinicians [34].

#### Smartphone Apps

Of the 21 studies, 6 (29%) based on smartphone apps directly distinguish between UD and BD. Among these, 5 (83%) studies [24,25,27-29] predominantly used the Monsenso system—a smartphone-based monitoring platform installed on patients’ personal devices (compatible with both iPhone and Android). The system gathered data through daily patient-reported entries, including subjective information such as mood, sleep, and activity levels. In addition, it automatically collected objective data from smartphone sensors, such as phone use patterns, mobility metrics, and voice features. These 5 studies were part of the RADMIS trials [45], a pragmatic, investigator-blinded, randomized controlled trial. In the intervention group, patients received the Monsenso system enabled patients to self-monitor their symptoms, access psychoeducational resources, and engage with cognitive modules. In contrast, the control group received standard treatment alone. The trial spanned 6 months, with outcome assessments conducted at baseline (0 months) and at 3 and 6 months. Another study [26] used the mindLAMP app, which collected real-time data, including geolocation, accelerometer readings, and screen-state (on or off) information. Participants received notifications 3 times per week, prompting them to complete in-app surveys measuring self-reported depression (Patient Health questionnaire-2) and anxiety (Generalized Anxiety Disorder 2-item). In contrast, the Monsenso system used a different scale for patient-reported, smartphone-based mood evaluation. For patients experiencing depressive states, mood scores ranged from −3 to −1, with a neutral mood (euthymia) defined as a self-reported score between −0.5 and +0.5. A total of 2 (33%) out of 6 studies, which used only participants’ daily active data from self-assessment questionnaires via smartphone apps, revealed that patients with UD spent a higher proportion of time with irritability compared to patients with BD (depressive state) [27]. In contrast, patients with BD exhibited a lower level of activity compared to those with UD (overall, euthymic state and depressive state) [29]. The remaining 4 (67%) out of 6 studies used various machine learning models. Of these 4 studies, 1 (25%) study [24] demonstrated that using a balanced bagging classifier with combined smartphone location data effectively distinguished BD from UD during both depressive and euthymic states, achieving a high AUC of 0.79. Another study [25] found that voice features could differentiate BD from UD with low sensitivity but relatively high specificity. A third study [26] used logistic regression to classify UD and BD, reporting a best test accuracy of 64.3% and a test AUC of 0.62. The final study [28] applied a random forest model using combined sensor-based smartphone data, where leave-one-out cross-validation yielded a sensitivity of 0.54, specificity of 0.44, and an AUC of 0.48.

#### Wearable Devices

Wearable devices or sensors are tools that monitor physiological and behavioral data, such as heart rate, movement, and temperature, enabling continuous, noninvasive tracking of health and activity patterns. Such tools were used in 3 (14%) out of 21 studies, with 2 (67%) studies classifying UD, BD, and HC, and 1 (33%) study directly distinguishing between UD and BD. In total, 2 (67%) studies used the Actiwatch, a lightweight wrist-worn accelerometer, to differentiate between UD and BD [30] or classify UD, BD, and HC [36]. The device recorded participants’ activity in 2-minute epochs over several weeks, capturing data on sleep or activity patterns. Another study [35] used the research-grade wearable device Empatica E4 to collect physiological data across multiple channels, including acceleration, skin temperature, blood volume pulse, heart rate, and electrodermal activity. Of the 3 studies, 1 (33%) study [30] used principal component analysis to identify distinct temporal patterns of intraday activities differentiating BD from UD, including significant (*p*<0.05) differences in activity patterns (eg, morning hyperactivity in BD vs morning hypoactivity in UD). Another small-sample study [36] achieved an accuracy of 0.991 and an *F*_1_-score of 0.9887 in a 4-class classification task (UD, BD type 1, BD type 2, and HC) using machine learning. The final study [35] used physiological data collected by the wearable device Empatica E4 to classify 7 groups, including different episodes of UD and BD, remission phases of both disorders, and HC, achieving an accuracy of 0.7 and an *F*_1_-score of 0.6927.

#### Audiovisual Recordings

Of 21 studies, 11 (52%) studies used audiovisual recordings, with 4 (36%) studies [31-34] directly distinguishing between UD and BD, while the remaining studies [37-43] focused on the UD, BD, and HC classification task. Among these, 5 (45%) studies [33,34,37,40,43] examined speech alone, 3 (27%) studies [38,39,42] investigated only facial expressions, 2 (18%) studies [31,41] combined speech with facial expressions, and 1 (9%) study [32] focused exclusively on body motion. Of 11 studies, 7 (64%) studies [31,37-42] were based on the CHI-MEI mood disorder database, but they differed in the types of data used or the machine learning classification methods applied. Unlike studies using smartphone apps or wearable devices or sensors, research involving audiovisual recordings typically collected participants’ speech, facial expressions, and even body motion within a single day using a fixed paradigm. Of 7 studies that included the speech modality, 4 (57%) [31,37,40,41] extracted emotion profiles, which represent the local intensity of emotions for each speech response corresponding to a specific question. These emotion profiles were used for single-modality analysis or integrated with facial expressions for fusion analysis in machine learning classification tasks. Among 5 studies that included the facial expression modality, 4 (80%) studies [31,39,41,42] analyzed facial action units. A total of 2 (40%) studies examined motion vectors [38,42], which were used to capture subtle changes in facial expressions at a microscopic level. Among the 4 (36%) studies that directly distinguished between UD and BD, a single study [32] explored the correlation between body motion and mood disorders, finding no significant differences in any head motion features between the UD and BD groups. The remaining 3 (75%) studies used machine learning methods or analyses of covariance to differentiate between the 2 groups. In the binary classification task, 1 (33%) study [34] reported both AUC and accuracy of 0.50, after extracting i-vectors from Mel-frequency cepstral coefficients. Another study, which combined facial expressions and speech for fusion analysis, achieved an optimal accuracy of 65.38% using the coupled hidden Markov model [31]. Furthermore, 1 (33%) study found that patients with UD exhibited longer response times compared to those with BD, although no significant differences were observed in speech rate or pause time [33]. For the remaining 7 (64%) studies, all used machine learning methods (eg, long short-term memory, random forest, and support vector machines) to classify UD, BD, and HC. The model accuracies ranged from 61.1% to 95.6%.

#### Multimodal Technology

Of 21 studies, only 1 (5%) study was categorized as “multimodal technology.” Although this study also collected digital phenotyping data via smartphones, its approach differed from the previously mentioned “Smartphone Apps” section (as it did not involve long-term data collection over weeks or even months). Therefore, it was categorized separately. This study developed a virtual assistant for automatic depression-level stratification on mobile devices [44]. The assistant actively engages users through voice-based dialogues and dynamically adjusts conversation content based on emotion perception. During the interaction, multimodal features, including text, audio, facial expressions, heart rate, and eye movement, are extracted to facilitate precise depression-level stratification. The study used a feature-level fusion framework to integrate 5 modalities with a deep neural network for classifying 5 groups, including UD (with mild, moderate, and severe levels), BD, and HC. Using outcome data from 168 participants, the experimental results demonstrated that the feature-level fusion of all 5 modalities achieved the highest overall accuracy of 90.26%.

### Quality Assessment

The results of the methodological quality assessment using the QUADAS-2 tool were presented in Multimedia Appendix 4. The risk of bias in the included studies is considerable and cannot be overlooked. A detailed table summarizing the QUADAS-2 scores for each study is provided in Multimedia Appendix 5 [24-44].

## Discussion

### Principal Findings

We conducted a systematic review of original articles from both journals and conference proceedings, investigating the use of portable or wearable digital tools for distinguishing between UD and BD, as well as for classifying UD, BD, and HC. A total of 21 articles were included, categorized into 4 main groups based on the type of digital tool assessed: (1) smartphone apps for collecting active data such as mood self-assessments or passive data, location, naturalistic phone calls, device rotation, and more; (2) wearable sensors, including the Actiwatch and the research-grade wearable device Empatica E4; (3) audiovisual recordings for analyzing speech characteristics, facial expressions, and upper body movements; and (4) multimodal technology, which combine text, audio, facial expressions, heart rate, and eye movement data. Overall, digital phenotyping data offer substantial opportunities for advancing the differential diagnosis of mood disorders. Despite certain methodological limitations, our findings highlight the potential of these digital technologies to provide more precise and objective support in diagnosing mood disorders. Certain features, such as activity levels captured through smartphone apps or wearable devices, emerged as potential markers for directly distinguishing between UD and BD. Individuals with BD typically exhibited lower activity levels compared to those with UD. Moreover, patients with BD tended to show higher activity levels in the morning and lower activity in the evening, whereas patients with UD displayed the opposite pattern. In addition, approaches leveraging speech modalities or integrating multiple modalities achieved better classification performance across the UD, BD, and HC groups, although the specific contributing features remain unclear. This uncertainty could be attributed to the complex and diverse nature of voice features, including strictly acoustic features (eg, speech rate, pause duration, and response time) [33], prosodic features (eg, pitch, energy, and formants) [43,46], and spectral features (eg, gamma-tone frequency Cepstral coefficients and Mel-frequency cepstral coefficients) [34,47].

Diagnostic confusion between BD and UD often occurs when patients are in a remitted or depressive state, as patients and their families may fail to recall previous manic or hypomanic episodes, making it challenging for clinicians to make an accurate diagnosis [24]. Of the 21 included studies, 6 (29%) included studies that used smartphone apps to directly distinguish between UD and BD. Of these, 5 (83%) studies using the Monsenso system considered the clinical staging of both groups and used different types of digital phenotyping data to differentiate between UD and BD. Among them, 3 (60%) studies used machine learning algorithms, with the best AUC of 0.79 achieved in both euthymic and depressive states (based on location data) [24]. However, results from 2 (40%) other studies [25,28] showed lower AUC values (0.42-0.58), which may indicate that variations in smartphone-based digital phenotyping are highly individualized [28]. In total, 4 (67%) [25-27,29] out of 6 studies used participants’ active data to distinguish between UD and BD, including daily self-assessments of irritability, mood, activity, sleep, stress, anxiety, and naturalistic phone calls voice. Half of these studies (2/4, 50%) used mixed-effects models, revealing differences in the level of activity [29] and presence of irritability [27] between UD and BD in a depressive state.

Interestingly, among the 3 (14%) out of 21 studies that used wearable devices to collect digital phenotyping data, 1 (33%) study also revealed differences in activity patterns between UD and BD. In contrast to the study mentioned earlier that used daily self-report activity scales, this study collected participants’ activity levels using activity trackers. This study provided a more detailed insight into the activity patterns of UD and BD: BD showed a high activity pattern in the morning and a low activity pattern in the evening, while UD exhibited the opposite [30]. Another study based on wearable watches also considered activity levels as a digital phenotype, but included HC as well. Using machine learning for a 4-class classification of UD, BD type 1, BD type 2, and HC, the study achieved an accuracy of 0.991 [36]. These 3 studies all demonstrate the potential of activity levels in the differential diagnosis of mood disorders.

Among the studies using audiovisual recordings and multimodal technologies to classify UD, BD, and HC, nearly all involved one-time data collection from participants in a standardized environment. Overall, approaches based on speech modalities or the integration of multiple modalities demonstrated better classification performance across these 3 groups, with accuracy ranging from 75.56% to 95.6% [37,40,41,43,44]. However, the specific features contributing to the classification performance remained unclear.

Accordingly, it can be concluded that smartphone apps and wearable devices, when combined with machine learning or other advanced analytical methods, demonstrate moderate diagnostic potential for differentiating UD and BD. However, their effectiveness is often limited by substantial individual variability in digital phenotyping data. Similarly, studies using audiovisual recordings and multimodal technology with machine learning have shown mixed outcomes, while they exhibit promising results in multiclass classification tasks, they have had limited success in directly distinguishing between UD and BD. These findings suggest that, although digital phenotyping holds potential for supporting the differential diagnosis of mood disorders, it should complement, rather than replace, comprehensive clinical evaluations conducted by trained professionals.

In particular, digital phenotyping data collected via smartphones or wearable devices or sensors may be more suitable for long-term dynamic monitoring and even intervention in the real world. This advantage lies in its ability to continuously gather active or passive data, offering a more comprehensive understanding of an individual’s mental and physical state. By capturing data in real time, these approaches help mitigate recall biases commonly associated with other symptom-reporting methods [48]. Moreover, they address the issue of “back-filling,” a frequent problem with paper-based diaries [49], thereby improving the reliability and accuracy of symptom tracking. However, factors such as patients’ proficiency in using electronic devices, the need for continuous and uninterrupted use, and potential limitations in monitoring continuity due to lack of feedback must be considered [50], as these could impact its medical value. In contrast, multimodal data collected through fixed paradigms in controlled settings offer advantages, such as capturing explicit behaviors (eg, speech, facial expressions, gestures) and implicit physiological signals (eg, eye movements, heart rate). Standardized protocols ensure reliable and reproducible data, making this structured approach particularly effective for mood disorder detection and classification. For instance, in the studies by Valstar et al [51] and Ringeval et al [52], audiovisual signals have been proven to play a substantial role in the detection and classification of UD and BD.

Our systematic review primarily focused on criterion validity and content validity. Criterion validity, defined as the extent to which the results of a specific test align with those of a reference standard [53], was demonstrated in the included studies by comparing classifications or distinctions based on digital phenotyping with diagnostic outcomes from professional medical evaluations. Content validity, on the other hand, refers to the degree to which an assessment tool is relevant to and representative of the targeted construct it aims to measure [54]. The included studies primarily focused on capturing one or a few dimensions of emotions (eg, daily mood, social interactions, or activity levels), falling short of comprehensively reflecting all core features of mood disorders. Furthermore, the complexity and vastness of digital phenotyping data led most studies to use machine learning algorithms for classification tasks, which posed substantial challenges for the evaluation of discriminant validity and structural validity.

### Future Directions

The article that first introduced the concept of digital phenotyping stated that data collected through social media, forums, online communities, wearable technologies, and mobile devices offers substantial value that goes beyond traditional methods such as laboratory tests and clinical imaging [11]. This is particularly substantial given the unclear pathogenesis, the unknown etiologies, and the lack of objective biomarkers of mood disorders. As a result, digital phenotyping stands out as a promising tool in supporting mood disorder diagnosis. We should also recognize that as medical technology advances, the types of digital phenotyping data are likely to expand. Beyond the digital devices discussed in our review, smart speakers may also play a role in future applications [55]. Besides, traditional laboratory tests and imaging methods are becoming more portable, including smartwatch-based 9-lead electrocardiograms [56], wearable electroencephalography [57], and even devices for long-term monitoring of metabolic indicators, such as continuous glucose monitors [58]. This evolution suggests that, in the future, more complex and extensive multimodal data may be used for the auxiliary diagnosis of mood disorders. However, the growing diversity of data types increases the demand for more sophisticated data analysis and processing techniques. To meet these challenges, integrating multimodal data from various sources could provide a more comprehensive understanding of mood disorders and expand the potential applications of digital phenotyping in mental health.

Despite the promise of digital phenotyping, we observed that this field is still relatively new, with a limited number of studies conducted thus far. Most of the research in this area involves small sample sizes and lacks consistency in evaluation standards and procedures. This makes it difficult to draw generalizable conclusions and underscores the need for further research to refine methodologies, improve data quality, and establish robust analytical frameworks. Therefore, it is crucial to develop standardized methodologies, guidelines, and protocols in the field of digital phenotyping to ensure consistency and reliability in data collection, analysis, and interpretation. Establishing these standards will enable better comparisons across studies, improve reproducibility, and ultimately enhance the clinical applicability of digital phenotyping for the diagnosis and monitoring of mood disorders.

Undoubtedly, digital phenotyping plays a substantial role in the prevention, diagnosis, treatment, and management of mood disorders and even mental disorders in general. However, several key requirements must be met to transform the way mental health care is delivered [59]. First, given the unique nature of mental disorders, patient privacy and data security require greater attention than for the general population. Patients must be fully informed and consent to how their data are collected, managed, and used, as this is crucial to maintaining trust between patients and clinicians [16]. Moreover, the “black box” nature of machine learning models continues to raise long-term concerns among health care professionals [60], as these models obscure their decision-making processes [12]. Such opacity limits the clinical actionability of machine learning models, particularly for classification tasks, as clinicians often require clear explanations to trust and act upon this prediction. This underscores the importance of explainable artificial intelligence [61], which aims to make the reasoning behind machine learning conclusions transparent, thus enhancing trust among health care professionals and ensuring that patients maintain greater control over their health data. However, the integration of digital tools into mental health care also faces broader social challenges. For example, these technological advances may exacerbate existing inequalities in access to psychiatric care, particularly disadvantaging underprivileged populations who lack access to the necessary technology or infrastructure [62]. Furthermore, the stigma surrounding mental illness [63], which already acts as a substantial barrier to seeking help, may be compounded by the perceived invasiveness of digital health tools, discouraging their adoption by those most in need.

### Strengths and Limitations

To the best of our knowledge, this is the first systematic review to comprehensively synthesize existing evidence on distinguishing BD from UD based on digital phenotyping. By systematically evaluating studies using smartphone apps, wearable devices, audiovisual recordings, and multimodal technologies, this review highlighted the emerging potential of digital phenotyping as a supportive tool for the differential diagnosis of mood disorders. However, our systematic review has several limitations. First, many of the included studies had small sample sizes, which increases the risk of overfitting and limits the generalizability of the findings. Future research should involve larger and more diverse samples to improve model robustness and applicability. Second, none of the studies validated their findings using external datasets, raising concerns about the reliability and reproducibility of the results. Incorporating independent datasets for external validation is essential to ensure consistent performance across different populations. Third, there was substantial heterogeneity in study designs, including variations in data sources, analytical methods, and outcome measures. This heterogeneity complicates direct comparisons and weakens the strength of synthesized conclusions. To address this, future studies should adopt standardized methodologies and outcome definitions to enhance comparability.

### Conclusions

In conclusion, our review shows that digital phenotyping for distinguishing UD and BD is progressing rapidly. However, challenges such as privacy, data security, and equitable access must be addressed for digital health care to effectively transform mental health care. Only by overcoming these challenges can digital innovations fulfill their potential to improve mental health care inclusivity.

## Appendix

Multimedia Appendix 1 PRISMA checklist.

Multimedia Appendix 2 Search strategy.

Multimedia Appendix 3 Demographic characteristics and diagnostic criteria of included studies.

Multimedia Appendix 4 Methodological quality assessment of the studies included in the systematic review through the Quality Assessment of Diagnostic Accuracy Studies-2.

Multimedia Appendix 5 Risk of bias in studies included in the systematic review: Quality Assessment of Diagnostic Accuracy Studies-2.

## Abbreviations

AUC: area under the curve
BD: bipolar disorder
HC: healthy control
PICOS: population, intervention, comparison, outcome, and study design
PRISMA: Preferred Reporting Items for Systematic reviews and Meta-Analyses
QUADAS-2: Quality Assessment of Diagnostic Accuracy Studies-2
UD: unipolar depression
