# Supplementary material for: Using Digital Phenotyping to Discriminate Unipolar Depression and Bipolar Disorder: Systematic Review
Source: J Med Internet Res. 2025 May 23;27:e72229. doi: 10.2196/72229 (PMC12144479; doi:10.2196/72229)
Supplement: Multimedia Appendix 1 [file jmir_v27i1e72229_app1.docx]

### **Table S1.** PRISMA (Preferred Reporting Items for Systematic reviews and Meta-Analyses) checklist

| **#** | **Section/topic** | **Checklist item and brief description of how the criteria were handled** | **Section, page** |
| --- | --- | --- | --- |
| **TITLE** | | | |
| 1 | Title | *Identify the report as a systematic review, meta-analysis, or both.*  The study has been identified as a systematic review of using digital phenotyping to discriminate unipolar depression and bipolar disorder. | Title |
| **ABSTRACT** | | | |
| 2 | Structured summary | *Provide a structured summary including, as applicable: background; objectives; data sources; study eligibility criteria, participants and interventions; study appraisal and synthesis methods; results; limitations; conclusions and implications of key findings; systematic review registration number.*  All relevant information has been included in the abstract. | Abstract |
| **INTRODUCTION** | | | |
| 3 | Rationale | *Describe the rationale for the review in the context of what is already known.*  The rationale for this review stems from the challenges clinicians face in distinguishing bipolar disorder (BD) from unipolar depression (UD), as both share overlapping symptoms. Traditional diagnostic methods rely heavily on self-report and clinical observation, which can be subjective. Digital phenotyping, leveraging real-time data from mobile devices and wearables, offers a promising, objective approach to identify behavioral patterns, potentially improving diagnostic accuracy and early differentiation between the two disorders. | Introduction, Background |
| 4 | Objectives | *Provide an explicit statement of questions being addressed with reference to participants, interventions, comparisons, outcomes, and study design (PICOS).*  This systematic review aims to achieve two goals: (1) to summarize the existing literature on the use of digital phenotyping to directly distinguish between UD and BD; (2) to review studies that utilize digital phenotyping to classify UD, BD, and healthy controls (HC). Furthermore, the review seeks to identify gaps in the current research and propose directions for future studies. | Introduction, Objectives |
| **METHODS** | | | |
| 5 | Protocol and registration | *Indicate if a review protocol exists, if and where it can be accessed (e.g., Web address), and, if available, provide registration information including registration number.*  The protocol is listed in the PROSPERO register (registration number: CRD42024624202). | Methods, Information Sources and Search Strategy |
| 6 | Eligibility criteria | *Specify study characteristics (e.g., PICOS, length of follow-up) and report characteristics (e.g., years considered, language, publication status) used as criteria for eligibility, giving rationale.*  We included original published articles written in English, with no restrictions on publication date.   - Population: patients diagnosed with UD, BD, and HC - Intervention: employment of a portable or wearable digital device, such as smartphone apps, wearable sensors, audio and/or visual recordings, and multimodal tools - Comparisons: a) digital phenotyping discrimination results vs diagnostic results through professional medical evaluation; b) UD vs BD; c) UD vs BD vs HC - Outcome: classification performances. If not available, results of t-tests/ANOVA analyses, non-parametric statistical tests, or correlation analyses. - Study design: original studies in English apart from review, narrative review, systematic review and meta-analysis   Articles were excluded if: a) were review, narrative review, systematic review and meta-analysis; b) were written in languages other than English; c) used technologies that were clearly unsuitable for daily monitoring; d) only included patients diagnosed with conditions other than UD and BD; e) had diagnoses not made through a professional medical evaluation (e.g., studies based on social media); f) were based on electronic health records or clinical data. | Methods, Eligibility Criteria |
| 7 | Information sources | *Describe all information sources (e.g., databases with dates of coverage, contact with study authors to identify additional studies) in the search and date last searched.*  Two-step search strategy: 1) Scopus, IEEE Xplore, PubMed, EMBASE, Web of Science and PsycINFO databases for articles published up to 20/3/2025. 2) electronic manual search of the reference lists of the retrieved articles. | Methods, Information Sources and Search Strategy |
| 8 | Search | *Present full electronic search strategy for at least one database, including any limits used, such that it could be repeated.*  The following terms were used: [(Depression OR depressive) AND (Bipolar OR mania) AND (“digital phenotyping” OR “digital phenotype” OR “digital biomarker” OR “digital footprint” OR “mobile sensing” OR “passive sensing” OR “computer vision techniques” OR “facial action unit” OR “speech analysis” OR “text analysis” OR “natural language processing” OR wearable OR portable OR sensor OR smartphone OR smart OR “mobile phone” OR “machine learning” OR “neural network” OR “artificial intelligence”)]. | Multimedia Appendix 2 |
| 9 | Study selection | *State the process for selecting studies (i.e., screening, eligibility, included in systematic review, and, if applicable, included in the meta-analysis).*  The identified articles were screened by title and abstract, and the full text of surviving articles were further inspected for eligibility against *a priori* defined inclusion and exclusion criteria. | Methods, Selection Process |
| 10 | Data collection process | *Describe method of data extraction from reports (e.g., piloted forms, independently, in duplicate) and any processes for obtaining and confirming data from investigators.*  Data extraction was performed by two independent researchers [RZ, XW]. Any discrepancy was discussed until a consensus was reached. Disagreements were resolved by a third reviewer (YF). | Methods, Data Extraction |
| 11 | Data items | *List and define all variables for which data were sought (e.g., PICOS, funding sources) and any assumptions and simplifications made.*  Extracted variables: geographic regions, population, epidemiological data of the sample (number and percentage of females, mean age), diagnostic criteria or psychiatric assessments, technological tool and data types details, duration of data collection, data preprocessing methods, specific variables/features selected, machine learning algorithms and/or statistical tests, validation (yes/no), main findings. | Methods, Data Extraction |
| 12 | Risk of bias in individual studies | *Describe methods used for assessing risk of bias of individual studies (including specification of whether this was done at the study or outcome level), and how this information is to be used in any data synthesis.*  Risk of bias was assessed independently by two reviewers (RZ, XW) through the Quality Assessment of Diagnostic Accuracy Studies-2 (QUADAS-2). | Methods, Study Risk of Bias Assessment |
| 13 | Summary measures | *State the principal summary measures (e.g., risk ratio, difference in means).*  The main findings of the individual studies were reported in. |  |
| 14 | Synthesis of results | *Describe the methods of handling data and combining results of studies, if done, including measures of consistency (e.g., I^2^) for each meta-analysis.*  The main findings of the individual studies were reported in Tables 1-2 and Multimedia Appendix 3. Data from individual studies could not be combined due to extreme heterogeneity. | Tables 1-2, Multimedia Appendix 3 |
| 15 | Risk of bias across studies | *Specify any assessment of risk of bias that may affect the cumulative evidence (e.g., publication bias, selective reporting within studies).*  The risk of bias and concerns regarding applicability were analysed for each domain of the QUADAS-2 and rated as a low, high or unclear risk. | Methods, Quality Assessment |
| 16 | Additional analyses | *Describe methods of additional analyses (e.g., sensitivity or subgroup analyses, metaregression), if done, indicating which were pre-specified.*  N/A | N/A |
| **RESULTS** | | | |
| 17 | Study selection | *Give numbers of studies screened, assessed for eligibility, and included in the review, with reasons for exclusions at each stage, ideally with a flow diagram.*  All details are depicted in the PRISMA flow-chart (Figure 1), and described in the main text. | Results, Figure 1 |
| 18 | Study characteristics | *For each study, present characteristics for which data were extracted (e.g., study size, PICOS, follow-up period) and provide the citations.*  For included studies, characteristics and citations are listed in Tables 1-2 and Multimedia Appendix 3. | Results, Tables 1-2, Multimedia Appendix 3 |
| 19 | Risk of bias within studies | *Present data on risk of bias of each study and, if available, any outcome level assessment (see item 12).*  Risk of bias is reported in the main text. Details are reported in Supplementary material (Multimedia Appendix 4 and Multimedia Appendix 5). | Results, Quality assessment, Multimedia Appendix 4 and Multimedia Appendix 5 |
| 20 | Results of individual studies | *For all outcomes considered (benefits or harms), present, for each study: (a) simple summary data for each intervention group (b) effect estimates and confidence intervals, ideally with a forest plot.*  N/A (no summary data due to high heterogeneity). The main findings of the individual studies are summarized in main text and reported in Tables 1-2. | Results, Tables 1-2 |
| 21 | Synthesis of results | *Present results of each meta-analysis done, including confidence intervals and measures of consistency.*  N/A | N/A |
| 22 | Risk of bias across studies | *Present results of any assessment of risk of bias across studies (see Item 15).*  Risk of bias is reported in the main text. Details are reported in Supplementary material (Multimedia Appendix 4 and Multimedia Appendix 5). | Results, Quality assessment, Multimedia Appendix 4 and Multimedia Appendix 5 |
| 23 | Additional analysis | *Give results of additional analyses, if done (e.g., sensitivity or subgroup analyses, meta-regression [see Item 16]).*  N/A | N/A |
| **DISCUSSION** | | | |
| 24 | Summary of evidence | *Summarize the main findings including the strength of evidence for each main outcome; consider their relevance to key groups (e.g., healthcare providers, users, and policy makers).* | Discussion |
| 25 | Limitations | *Discuss limitations at study and outcome level (e.g., risk of bias), and at review-level (e.g., incomplete retrieval of identified research, reporting bias).* | Discussion |
| 26 | Conclusions | *Provide a general interpretation of the results in the context of other evidence, and implications for future research.* | Discussion |
| **FUNDING** | | | |
| 27 | Funding | *Describe sources of funding for the systematic review and other support (e.g., supply of data); role of funders for the systematic review.*  This work was financially supported by the Funding Project of Clinical Research Center of Shanghai Mental Health Center Key Project (CRC2021DX01). | Acknoledgments |
