# Supplementary material for: Using Digital Phenotyping to Discriminate Unipolar Depression and Bipolar Disorder: Systematic Review
Source: J Med Internet Res. 2025 May 23;27:e72229. doi: 10.2196/72229 (PMC12144479; doi:10.2196/72229)
Supplement: Multimedia Appendix 2 [file jmir_v27i1e72229_app2.docx]

### **Table S2.** Search strategy

| Database | Search query | Items founded |
| --- | --- | --- |
| Pubmed | "Depressive Disorder"[Mesh] AND "Bipolar Disorder"[Mesh] AND (“digital phenotyping” OR “digital phenotype” OR “digital biomarker” OR “digital footprint” OR “mobile sensing” OR “passive sensing” OR “computer vision techniques” OR “facial action unit” OR “speech analysis” OR “text analysis” OR “natural language processing” OR wearable OR portable OR sensor OR smartphone OR smart OR “mobile phone” OR “machine learning” OR “neural network” OR “artificial intelligence”) | 114 |
| IEEE Xplore | (Depression OR depressive) AND (Bipolar OR mania) AND (“digital phenotyping” OR “digital phenotype” OR “digital biomarker” OR “digital footprint” OR “mobile sensing” OR “passive sensing” OR “computer vision techniques” OR “facial action unit” OR “speech analysis” OR “text analysis” OR “natural language processing” OR wearable OR portable OR sensor OR smartphone OR smart OR “mobile phone” OR “machine learning” OR “neural network” OR “artificial intelligence”)  Filters Applied: Conferences Journals | 231 |
| EMBASE | #1  'major depression'/exp AND 'bipolar disorder'/exp AND (‘digital phenotyping’:ti,ab,kw OR ‘digital phenotype’:ti,ab,kw OR ‘digital biomarker’:ti,ab,kw OR ‘digital footprint’:ti,ab,kw OR ‘mobile sensing’:ti,ab,kw OR ‘passive sensing’:ti,ab,kw OR ‘computer vision techniques’:ti,ab,kw OR ‘facial action unit’:ti,ab,kw OR ‘speech analysis’:ti,ab,kw OR ‘text analysis’:ti,ab,kw OR ‘natural language processing’:ti,ab,kw OR wearable:ti,ab,kw OR portable:ti,ab,kw OR sensor:ti,ab,kw OR smartphone:ti,ab,kw OR smart:ti,ab,kw OR ‘mobile phone’:ti,ab,kw OR ‘machine learning’:ti,ab,kw OR ‘neural network’:ti,ab,kw OR ‘artificial intelligence’:ti,ab,kw)  #1 AND ('article'/it OR 'conference abstract'/it) | 356 |
| Scopus | ( TITLE-ABS-KEY ( depression ) OR TITLE-ABS-KEY ( depressive ) ) AND ( TITLE-ABS-KEY ( "bipolar disorder" ) OR TITLE-ABS-KEY ( mania ) ) AND ( TITLE-ABS-KEY ( "digital phenotyping" ) OR TITLE-ABS-KEY ( "digital phenotype" ) OR TITLE-ABS-KEY ( "digital biomarker" ) OR TITLE-ABS-KEY ( "digital footprint" ) OR TITLE-ABS-KEY ( "mobile sensing" ) OR TITLE-ABS-KEY ( "passive sensing" ) OR TITLE-ABS-KEY ( "computer vision techniques" ) OR TITLE-ABS-KEY ( "facial action unit" ) OR TITLE-ABS-KEY ( "speech analysis" ) OR TITLE-ABS-KEY ( "text analysis" ) OR TITLE-ABS-KEY ( "natural language processing" ) OR TITLE-ABS-KEY ( wearable ) OR TITLE-ABS-KEY ( portable ) OR TITLE-ABS-KEY ( sensor ) OR TITLE-ABS-KEY ( smartphone ) OR TITLE-ABS-KEY ( smart ) OR TITLE-ABS-KEY ( "mobile phone" ) OR TITLE-ABS-KEY ( "machine learning" ) OR TITLE-ABS-KEY ( "neural network" ) OR TITLE-ABS-KEY ( "artificial intelligence" ) ) AND ( LIMIT-TO ( LANGUAGE , "English" ) ) AND ( LIMIT-TO ( DOCTYPE , "ar" ) OR LIMIT-TO ( DOCTYPE , "cp" ) ) | 982 |
| Web of Science | TS=((Depression OR depressive) AND (Bipolar OR mania) AND (“digital phenotyping” OR “digital phenotype” OR “digital biomarker” OR “digital footprint” OR “mobile sensing” OR “passive sensing” OR “computer vision techniques” OR “facial action unit” OR “speech analysis” OR “text analysis” OR “natural language processing” OR wearable OR portable OR sensor OR smartphone OR smart OR “mobile phone” OR “machine learning” OR “neural network” OR “artificial intelligence”))  Document Types: Article or Proceeding Paper  Language：English | 782 |
| PsycINFO | SU(depression OR depressive) AND SU(bipolar OR mania) AND SU("digital phenotyping" OR "digital phenotype" OR "digital biomarker" OR "digital footprint" OR "mobile sensing" OR "passive sensing" OR "computer vision techniques" OR "facial action unit" OR "speech analysis" OR "text analysis" OR "natural language processing" OR wearable OR portable OR sensor OR smartphone OR smart OR "mobile phone" OR "machine learning" OR "neural network" OR "artificial intelligence")  Document Types: Academic theoretical journal  Language：English | 90 |
