# Supplementary material for: Using Digital Phenotyping to Discriminate Unipolar Depression and Bipolar Disorder: Systematic Review
Source: J Med Internet Res. 2025 May 23;27:e72229. doi: 10.2196/72229 (PMC12144479; doi:10.2196/72229)
Supplement: Multimedia Appendix 3 [file jmir_v27i1e72229_app3.docx]

### **Table S3.** **Demographic characteristics and diagnostic criteria of included studies that directly distinguish between UD^a^ and BD^b^.**

| Study | Region | Population n (%) | | Female n (%) | | Age (y), mean (SD) | | Diagnostic criteria or psychiatric assessments |
| --- | --- | --- | --- | --- | --- | --- | --- | --- |
|  |  | UD | BD | UD | BD | UD | BD |  |
| **Smartphone apps** | | | | | | | | |
| Faurholt-Jepsen et al [24], 2022 | Denmark | 75 (53.6) | 65 (46.4) | 39 (52) | 39 (60) | 44.3 (14.5) | 44.1 (13.27) | ICD^c^-10 |
| Faurholt‐Jepsen et al [25], 2022 | Denmark | 48 (28.4) | 121 (71.6) | 29 (60) | 73 (60.3) | 45.6 (14.9) | 35.7 (12.3) | ICD-10 |
| Langholm et al [26], 2023 | United States | 53 (65) | 28 (35) | —^d^ | — | — | — | MINI^e^ |
| Faurholt-Jepsen et al [27], 2023 | Denmark | 58 (15.5) | 316 (84.5) | 28 (48) | 214 (67.7) | 43.34 (14.1) | 38.5 (10.8) | ICD-10 |
| Faurholt-Jepsen et al [28], 2024 | Denmark | 74 (53.6) | 64 (46.4) | 39 (53) | 39 (61) | 44.3 (14.5) | 44.1 (13.27) | ICD-10 |
| Faurholt-Jepsen et al [29], 2025 | Denmark | 59 (55.7) | 47 (44.3) | 28 (47) | 24 (51) | 44.5 (14) | 41.6 (13.3) | ICD-10 |
| **Wearable devices** | | | | | | | | |
| Tanaka et al [30], 2018 | Japan | 59 (63) | 35 (37)  (depression) | 41 (69) | 12 (34) | 53.7 (18.6) | 46.9 (20.5) | DSM^f^-IV |
| **Audiovisual recordings** | | | | | | | | |
| Yang et al [31], 2016 | Taiwan | 13 (50) | 13 (50) | — | — | — | — | — |
| Horigome et al [32], 2020 | Japan | 17 (55) | 14 (45) | 9 (53) | 7 (50) | 57 (13.2) | 61 (20.9) | DSM-5 |
| Yamamoto et al [33], 2020 | Japan | 84 (55.3) | 68 (44.7) | 47 (56) | 38 (56) | 50 (15) | 55.1 (17) | DSM-5 |
| Pan et al [34], 2023 | China | 22 (50)  (actively symptomatic state) | 22 (50)  (euthymic state) | 11 (50) | 11 (50) | 34.18 (9.67) | 29.68 (8.99) | DSM-IV |

^a^UD: unipolar depression.

^b^BD: bipolar disorder.

^c^ICD: International Classification of Diseases.

^d^Not available.

^e^MINI: Mini International Neuropsychiatric Interview.

^f^DSM: Diagnostic and Statistical Manual of Mental Disorders.

### **T****able S4. Demographic characteristics and diagnostic criteria of included studies that** **classify UD^a^, BD^b^, and HC^c^.**

| Study | Region | Population n (%) | | | Female n (%) | | | Age (y), mean (SD) or range | | | Diagnostic criteria or psychiatric assessments |
| --- | --- | --- | --- | --- | --- | --- | --- | --- | --- | --- | --- |
|  |  | UD | BD | HC | UD | BD | HC | UD | BD | HC |  |
| **Wearable devices** | | | | | | | | | | | |
| Anmella et al [35], 2023 | Spain | 2 (11)  (euthymic); 2 (11)  (depressed episode) | 2 (11)  (euthymic);  2 (11)  (depressed episode);  2 (11)  (manic episode);  2 (11)  (mixed episode) | 7 (37) | 1 (50)  (euthymic); 0  (depressed episode) | 0  (euthymic);  0  (depressed episode);  0  (manic episode);  1 (50)  (mixed episode) | 4 (57) | 60  (euthymic); 51  (depressed episode) | 57.5  (euthymic);  34.5  (depressed episode);  30.5  (manic episode);  35  (mixed episode) | 31  (1.85) | DSM^d^-5 |
| Zakariah and Alotaibi [36], 2023 | Norway | 15 (27) | 1 (2)  (BD-1^e^ depression);  7 (13)  (BD-2^f^ depression) | 32 (58) | 7 (47) | 0  (BD-1);  3 (43)  (BD-2) | 20 (63) | 25-69 | 40-44  (BD-1);  20-64  (BD-2) | 20-69 | DSM-IV |
| **Audiovisual recordings** | | | | | | | | | | | |
| Yang et al [37], 2016 | Taiwan | 13 (33) | 13 (33) | 13 (33) | —^g^ | — | — | — | — | — | — |
| Su et al [38], 2017 | Taiwan | 12 (33) | 12 (33) | 12 (33) | — | — | — | — | — | — | — |
| Hong et al [39], 2018 | Taiwan | 12 (33) | 12 (33) | 12 (33) | — | — | — | — | — | — | — |
| Huang et al [40], 2019 | Taiwan | 15 (33) | 15 (33) | 15 (33) | 11 (73) | 10 (67) | 10 (67) | 46.4 (11.12) | 45.8 (10.03) | 31.6 (8.00) | — |
| Su et al [41], 2020 | Taiwan | 13 (33) | 13 (33) | 13 (33) | 27 (69) | | | — | — | — | — |
| Hong et al [42], 2021 | Taiwan | 12 (33) | 12 (33) | 12 (33) | — | — | — | — | — | — | — |
| Luo et al [43], 2024 | China | 50 (33.3) | 50 (33.3) | 50 (33.3) | 32 (64) | 37 (74) | 29 (58) | 14.48 (1.752) | 14.24 (1.585) | 14.55 (2.661) | DSM-5 |
| **Multimodal technology** | | | | | | | | | | | |
| Wu et al [44], 2024 | Taiwan | 31 (18.5)  (mild);  48 (28.6)  (moderate);  46 (27.4)  (severe) | 25 (14.9) | 18 (10.7) | 21 (68)  (mild);  43 (90)  (moderate);  38 (83)  (severe) | 22 (88) | 3 (17) | 14.88 (1.79)  (mild); 15.58 (1.36) (moderate); 14.96 (1.7) (severe) | 15.23 (1.47) | 23.67 (1.33) | ICD^h^-10 |

^a^UD: unipolar depression.

^b^BD: bipolar disorder.

^c^HC: healthy control.

^d^DSM: Diagnostic and Statistical Manual of Mental Disorders.

^e^BD-1: bipolar disorder type I.

^f^BD-2: bipolar disorder type II.

^g^Not available.

^h^ICD: International Classification of Diseases.
