# Supplementary material for: Using Digital Phenotyping to Discriminate Unipolar Depression and Bipolar Disorder: Systematic Review
Source: J Med Internet Res. 2025 May 23;27:e72229. doi: 10.2196/72229 (PMC12144479; doi:10.2196/72229)

### **Figure S1.** Methodological quality assessment of the studies included in the systematic review through the QUADAS-2


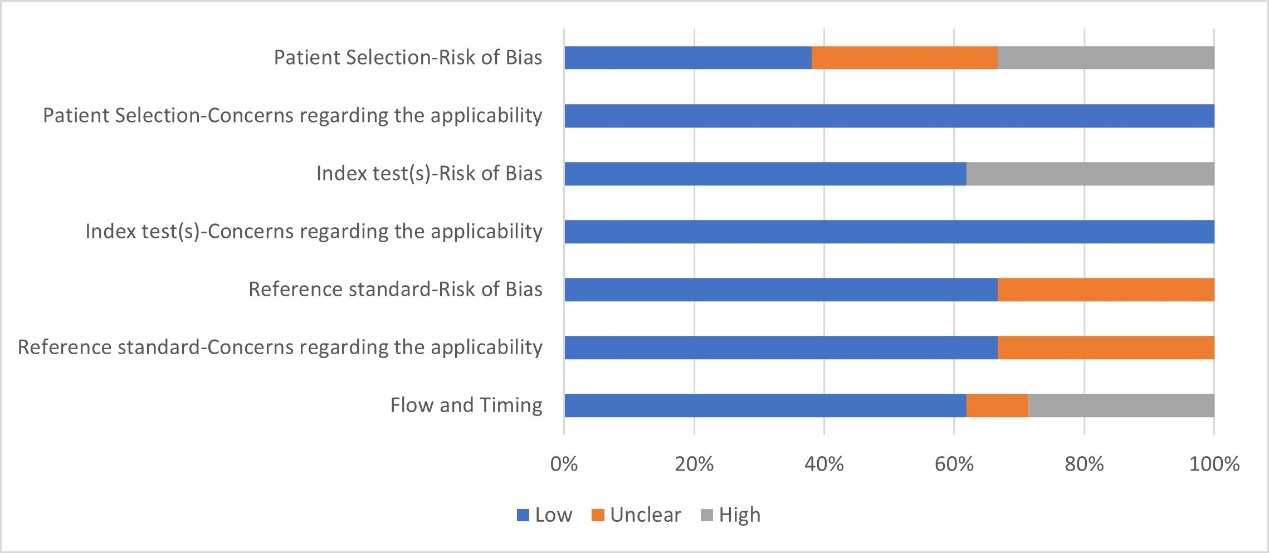

Supplement: Multimedia Appendix 4 [file jmir_v27i1e72229_app4.docx]
