# Supplementary material for: Using Digital Phenotyping to Discriminate Unipolar Depression and Bipolar Disorder: Systematic Review
Source: J Med Internet Res. 2025 May 23;27:e72229. doi: 10.2196/72229 (PMC12144479; doi:10.2196/72229)
Supplement: Multimedia Appendix 5 [file jmir_v27i1e72229_app5.docx]

### **Table S5.** Risk of bias in studies included in the systematic review: Quality Assessment of Diagnostic Accuracy Studies-2 (QUADAS-2)

|  | | **Patient Selection** | | **Index test(s)** | | **Reference standard** | | **Flow and Timing** |
| --- | --- | --- | --- | --- | --- | --- | --- | --- |
|  |  | *Risk of Bias* | *Concerns regarding the applicability* | *Risk of Bias* | *Concerns regarding the applicability* | *Risk of Bias* | *Concerns regarding the applicability* | *Risk of Bias* |
| **Smartphone app** | Faurholt-Jepsen et al [24], 2022 | 0 | 0 | 1 | 0 | 0 | 0 | 1 |
|  | Faurholt‐Jepsen et al [25], 2022 | 1 | 0 | 1 | 0 | 0 | 0 | 1 |
|  | Langholm et al [26], 2023 | 1 | 0 | 1 | 0 | 0 | 0 | 1 |
|  | Faurholt-Jepsen et al [27], 2023 | 1 | 0 | 1 | 0 | 0 | 0 | 1 |
|  | Faurholt-Jepsen et al [28], 2024 | 0 | 0 | 1 | 0 | 0 | 0 | 1 |
|  | Faurholt-Jepsen et al [29], 2025 | 0 | 0 | 1 | 0 | 0 | 0 | 1 |
| **Wearable device** | Tanaka et al [30], 2018 | 1 | 0 | 1 | 0 | 0 | 0 | 0 |
|  | Anmella et al [35], 2023 | 1 | 0 | 0 | 0 | 0 | 0 | 0 |
|  | Zakariah and Alotaibi [36], 2023 | 1 | 0 | 1 | 0 | 0 | 0 | 0 |
| **Audiovisual recordings** | Yang et al [37], 2016 | 5 | 0 | 0 | 0 | 5 | 5 | 0 |
|  | Yang et al [31], 2016 | 5 | 0 | 0 | 0 | 5 | 5 | 0 |
|  | Su et al [38], 2017 | 5 | 0 | 0 | 0 | 5 | 5 | 0 |
|  | Hong et al [39], 2018 | 5 | 0 | 0 | 0 | 5 | 5 | 0 |
|  | Huang et al [40], 2019 | 0 | 0 | 0 | 0 | 5 | 5 | 0 |
|  | Horigome et al [32], 2020 | 0 | 0 | 0 | 0 | 0 | 0 | 5 |
|  | Yamamoto et al [33], 2020 | 0 | 0 | 0 | 0 | 0 | 0 | 0 |
|  | Su et al [41], 2020 | 5 | 0 | 0 | 0 | 5 | 5 | 0 |
|  | Hong et al [42], 2021 | 5 | 0 | 0 | 0 | 5 | 5 | 0 |
|  | Pan et al [34], 2023 | 0 | 0 | 0 | 0 | 0 | 0 | 5 |
|  | Luo et al [43], 2024 | 0 | 0 | 0 | 0 | 0 | 0 | 0 |
| **Multimodal** | Wu et al [44], 2024 | 1 | 0 | 0 | 0 | 0 | 0 | 0 |

**0 = Low Risk；1 = High Risk；5 = Unclear Risk.**
